# Re-Evaluation of *Sinocastor* (Rodentia: Castoridae) with Implications on the Origin of Modern Beavers

**DOI:** 10.1371/journal.pone.0013990

**Published:** 2010-11-15

**Authors:** Natalia Rybczynski, Elizabeth M. Ross, Joshua X. Samuels, William W. Korth

**Affiliations:** 1 Earth Sciences, Canadian Museum of Nature, Ottawa, Canada; 2 Earth Sciences, Carleton University, Ottawa, Canada; 3 John Day Fossil Beds National Monument, Kimberly, Oregon, United States of America; 4 Rochester Institute of Vertebrate Paleontology, Rochester, New York, United States of America; University College London, United Kingdom

## Abstract

The extant beaver, *Castor*, has played an important role shaping landscapes and ecosystems in Eurasia and North America, yet the origins and early evolution of this lineage remain poorly understood. Here we use a geometric morphometric approach to help re-evaluate the phylogenetic affinities of a fossil skull from the Late Miocene of China. This specimen was originally considered *Sinocastor*, and later transferred to *Castor*. The aim of this study was to determine whether this form is an early member of *Castor*, or if it represents a lineage outside of *Castor*. The specimen was compared to 38 specimens of modern *Castor* (both *C. canadensis* and *C. fiber*) as well as fossil specimens of *C. fiber (Pleistocene)*, *C. californicus* (Pliocene) and the early castorids *Steneofiber eseri (early Miocene)*. The results show that the specimen falls outside the *Castor* morphospace and that compared to *Castor*, *Sinocastor* possesses a: 1) narrower post-orbital constriction, 2) anteroposteriorly shortened basioccipital depression, 3) shortened incisive foramen, 4) more posteriorly located palatine foramen, 5) longer rostrum, and 6) longer braincase. Also the specimen shows a much shallower basiocciptal depression than what is seen in living *Castor*, as well as prominently rooted molars. We conclude that *Sinocastor* is a valid genus. Given the prevalence of apparently primitive traits, *Sinocastor* might be a near relative of the lineage that gave rise to *Castor*, implying a possible Asiatic origin for *Castor*.

## Introduction

The fossil record of beavers (Castoridae) comprises roughly 30 genera, with the earliest representatives appearing in the latest Eocene of North America. By the early Oligocene the group had achieved a Holarctic distribution [1] and toward the end of the Oligocene at least three specialized lineages were represented. Two lineages, represented by *Migmacastor* and *Palaeocastor* among others, are fossorial specialists of relatively small body size (20–30 cm long) characterized by craniodental adaptations for digging, massive forelimbs, an enlarged manus, and a shortened neck and tail [2], [3], [4], [5], [6], [7], [8]. The third lineage exhibits skeletal proportions that indicate swimming specialization, specifically hind-limb paddling locomotion [3], [6], [7]. This group of semi-aquatic beavers is represented by species spanning a wide range of body sizes, and includes the Pleistocene giant beaver, *Castoroides*, which is over 2 m in length [6]. The semi-aquatic group diversified greatly in the Miocene. In this epoch there were at least ten genera in North America: *Castor, Dipoides, Anchitheriomys*, *Dipoides, Monosaulax*, *Eucastor* [*6]*, [*7*] *Nothodipoides* [*9*], *Prodipoides* [*10*], *Priusaulax* [11] and *Temporocastor* [12]. Except for *Castor*, these beavers were generally small bodied. One lineage within the semi-aquatic clade represented by *Eucastor, Nothodipoides and Temporocastor*, shows some cranial evidence consistent with tooth-digging. In Miocene Europe the semiaquatic group includes at least six genera: *Castor*, *Dipoides*, *Anchitheriomys*, *Steneofiber*, *Chalicomys*, *Euroxenomys* [13]. *Castor* is reported to have appeared in Europe, North America, and Asia in the Late Miocene [13], [14], [15], [16], however the results of this study suggest that these Asian “Castor” specimens are likely attributable to *Sinocastor*.

**Figure 1:**
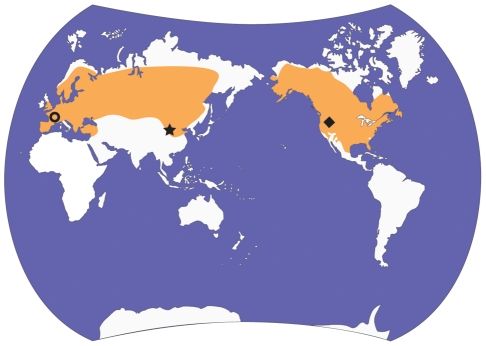
Map showing late Pleistocene distribution of *Castor* [**9**] and location of fossils used in this study. *Castor canadensis* and *C. fiber* are the only extant species, and are known from North America and Eurasia, respectively (orange shading). The locality of the fossil beaver under study, IVPP v-10471 (Also IVPP v-10472 and FAM 64072) is shown with a star. The localities for the *Steneofiber* and *C. californicus* material are shown with a circle and diamond shape, respectively.

Today *Castor* is represented by two species, the Eurasian *C. fiber* [17] and the North American *C. canadensis* [18]. Both species were formerly very abundant and the historic ranges of *C. fiber* and *C. canadensis* were among the largest for mammals; for example, the natural range of *C. canadensis* extends from North America's northern tree line to the southern United States and northern Mexico [19] (Fig. 1). The population size in the 1980's was estimated to be 6–12 million, whereas prior to exploitation of the European fur trade populations were estimated to be 60–400 million [20]. The success of *Castor* may at least be partly attributed to its ability to create and modify its habitat, resulting from a complex of behaviours associated with construction and food storage. *Castor* uses trees, which it fells, mud and other materials to build lodges and dams. The pond habitat created by *Castor* serves multiple functions, including maintaining the lodge's submerged entrance and providing an underwater storage for the foodpile. The foodpile comprises branches and is used in the winter as the primary foodsource [21]. The ecological consequences of *Castor*'s construction behaviour are profound and the genus is considered to be an ecosystem engineer and specialized niche constructor [21], [22], [23]. Beaver ponds and the subsequent successional communities (e.g., beaver meadows) differ in composition and functioning from their unmodified counterparts [22]. Also, by converting streams into ponds, beavers influence landscape processes including drainage and sedimentation patterns [24], [25]. It has been suggested that in some cases beaver ponds may be maintained over centuries [25].

This study is part of a research effort that aims to trace the evolutionary history of this ecologically important lineage. Here, we evaluate the taxonomic position of a contentious castorid specimen from the Late Miocene of Asia. The specimen in question is a near complete skull that is similar in size and hypsodonty to the modern *Castor*, but shows a combination of traits, including “primitive traits”, which led previous researchers to suggest the specimen belonged to a separate genus, *Sinocastor*. The specimen and related material have since been referred to *Castor*, with little explanation (see “Historical review” below). Here we redescribe the skull, and also use a geometric morphometric approach to evaluate its phylogenetic and taxonomic position.

### Historical review of *Sinocastor*

Young [26] first named the genus *Sinocastor* for three late Neogene castorid species from Mongolia and China, *S. anderssoni* [27] *S. zdanskyi* [28], and *S. broilii* [29], the latter being designated as the type species. He separated the new genus from *Castor* based on: 1) a slightly more rounded cross-sectional shape of the upper incisor, 2) stronger postorbital constriction of the skull, 3) presence of a masseteric fossa, and 4) paraflexus and hypoflexus on P4 alternating (“First internal and external folds of P4 alternating”) [26]. He also noted that the striids and striae were long, “reaching probably the base of the crown” [26].

In his review of beavers of the world, Stirton [30] included all of the species previously referred to *Sinocastor* in *Castor*. Young's genus was only mentioned in a footnote (page 447) and the validity of the genus was not discussed. Teilhard de Chardin [31] reviewed the castorids of the Pliocene and Pleistocene of China, and referred the Asian species to *Sinocastor*, but did not list a type species for the genus or include a discussion of *S. broilii*. He referred additional material to *S. anderssoni* and distinguished *Sinocastor* from *Castor* entirely on the morphology of the cheek teeth (rooted with shorter striae (-ids)). The next review of castorids from China was by Xu [15]. He included both *S. anderssoni* and *S. zdanskyi* in *Castor* without discussion, and listed *S. broilii* (or *broili*) as a junior synonym of *S. anderssoni*. Xu also suggested that *S. zdanskyi* was a likely synonym of *S. anderssoni*, but was known from too few specimens for complete comparison. Since Xu's [15] synonymy of *S. broilii* under *S. anderssoni*, the latter is now the designated type species of *Sinocastor*. In their classification of mammals, McKenna and Bell [32] listed *Sinocastor* as a junior synonym of *Castor* as well.

In 2006, IVPP v-10471, formerly considered the type specimen for *Sinocastor broilii*, became available for re-examination. Here, we examine the morphology of this specimen in detail and compare it to extant members of the genus *Castor* and two extinct species of the family. In order to better assess the morphological similarity and phenetic affinities of these species, we use a geometric morphometric approach.

### Geometric morphometrics and taxonomy

Researchers who study living taxa have available to them a variety of data, including molecular data, that can be used to identify discontinuities between populations and ultimately distinguish taxa, such as species. Taxonomy of extinct vertebrates can be more challenging because taxonomic decisions are usually based solely on patterns of morphological variation of the skeletal system. Moreover the fossil record may preserve species intermediates. In a stratigraphic section where two fossil species are found to be linked by fossil intermediates it may be possible to identify breaks in stratigraphic horizons that can be used as a taxonomic separation boundary [33]. For example, the species break may be chosen to coincide with a geological unconformity [34]. For fossil taxa with living near-relatives it is useful to designate species in relation to variation seen in the modern form [35], [36]. Thus a fossil may be designated a new taxon if it falls outside the modern morphospace.

Here we use geometric morphometrics to compare the cranial morphology of the fossil IVPP v-10471, originally designated *Sinocastor broilii* [29], to both living *Castor* species. Also included in the analysis are the extinct *Castor californicus*, known from the late Miocene and Pliocene of North America, and *Steneofiber castorinus*, from the early Miocene of Europe. Geometric morphometric analyses quantify the shape of a specimen using a series of two-dimensional or three-dimensional landmarks. These methods have been applied to many studies of ontogeny, functional morphology, and evolution [2], [37], [38], [39], [40], [41], [42], [43], [44], [45]. They have also been used in studies of phenetic affinities and phylogenetic relatedness [39], [46], [47], [48], [49]. In this study we evaluate whether the overall skull shape of IVPP v-10471 falls within the range of variation of modern *Castor* species. If it does not, the finding would provide support for the suggestion that the specimen be regarded as a member of a genus outside of *Castor*. The morphometrics results, combined with consideration of other traits such as shape of basicranial depression and tooth root morphology, may have implications for our understanding of the origins of *Castor*.

## Materials and Methods

### Institutional Abbreviations

AMNH, American Museum of Natural History (New York, USA); CMNMA, Canadian Museum of Nature, Mammal collections (Ottawa Canada); FAM Frick Collection, American Museum of Natural History (New York, USA); FMNH, Field Museum of Natural History, (Chicago, Illinois, USA); HAFO, Hagerman Fossil Beds National Monument (Hagerman, Idaho, USA); IMNH, Idaho Museum of Natural History (Pocatello, Idaho, USA); IVPP, Institute of Vertebrate Paleontology and Paleoanthropology, Academia Sinica (Beijing, China); MNHN, Muséum National D'Histoire Naturelle (Paris, France); NMNH, National Museum of Natural History, Smithsonian Institution (Washington, D.C.).

### Specimens

Fossil specimens used in the study– IVPP v-10471(C/9), is a nearly complete skull lacking dorsal part of the rostum, zygomatic arches and mandibles. Collected in 1929 in Northern China from Paote, Shanxi Province (Pontian Red Clays of Baode, locality 108 of Andersson and Zdansky), this specimen was originally described as the type specimen for *S. broilii* [29]. FAM 64072 is the anterior portion of a skull, and includes all cheek teeth. It was collected (Dec, 1933) in the same region as IVPP v-10471, if not from the same locality.

#### Extant beavers

A sample of 33 *Castor canadensis* and five *C. fiber*, representing a wide geographic range, was used for this comparison (Table S1). Only adult specimens were selected, identified by the presence of complete or nearly complete fusion between the basioccipital and basisphenoid [50].

#### Additional fossil material considered

Three specimens of *Castor californicus* from the Hagerman Fossil Beds (Pliocene of Idaho) included NMNH 26154, IMNH 84010, and HAFO 2243. A Pleistocene specimen of *Castor fiber*, FMNH UC1537, was used from Burwell Fen, Cambridgeshire in England. The early Miocene *Steneofiber* was represented by the near-complete skull of *S. castorinus*, MNHN SG 3654, from the type locality Saint Gérand-le-Puy (MN 2) in France. Images for the analysis of this specimen were from Stefen [51].

### Selection and imaging landmarks

The skulls for analysis were photographed in dorsal, ventral, and lateral views with a digital camera from a distance of 190 cm (to minimize parallax) and saved in JPEG format. In dorsal and ventral views, sandbags were used to hold the skull so that the palatal surface was parallel with the focal plane. In lateral view, the midsagittal plane of the skull was aligned parallel to the focal plane. Most specimens were about 1000 pixels long and the linearity of photographs was tested at all magnification levels used.

**Figure 2:**
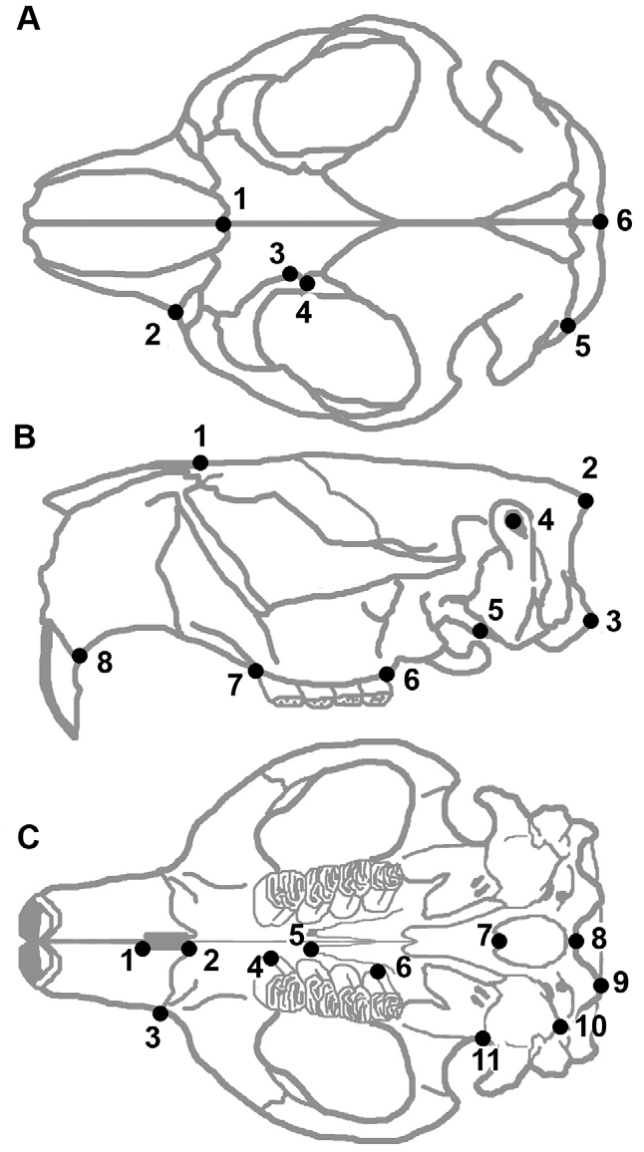
Morphological landmarks used in analysis. Landmarks are shown on a skull of *Castor canadensis* in dorsal (A), lateral (B) and ventral (C) view. Landmarks are described in Table 1.

The morphometrics program tpsDig2 [52] was used to digitize landmarks on each photograph. Twenty five landmarks (see Table 1, Fig. 2) were selected in three views with the aim of capturing the overall skull shape. The landmarks used were modified from those used in other recent studies of rodents [2], [40], [41], [53], [54]. For a given view the landmarks are located in roughly the same plane, and were selected so that they could be identified on different species [55]. To minimize the influence of possible asymmetry only one side was digitized for the dorsal and ventral views of the skull.

**Table 1:** List of landmarks used in geometric morphometric analyses and their descriptions (modified from 8, 41).

Landmark #	Description
Dorsal Cranium:	
1	Meeting point between the nasal and frontal along the midsagittal plane
2	Anterior tip of the suture between the premaxilla and the maxilla
3	Supraorbital notch
4	Tip of the postorbital process of the frontal
5	Y-shaped suture where the squamosal, parietal and occipital meet
6	Most posterior point of the interparietal along the midsagittal plane (meeting of sagittal and nuchal crests)
Lateral Cranium:	
1	Meeting point of the nasal and frontal along the midsagittal plane
2	Most posterior point of the interparietal along the midsagittal plane (meeting of sagittal and nuchal crests)
3	Most posterior point of the occipital condyle
4	External auditory meatus
5	Most ventral meeting point between the tympanic bulla and alisphenoid
6	Posterior end of tooth row
7	Anterior end of tooth row
8	Most posterior point of the alveolar rim
Ventral Cranium:	
1	Anterior tip of the incisive foramen
2	Posterior tip of the incisive foramen
3	Anterior tip of the infraorbital foramen
4	Meeting point between the P^4^ hypoflexus and alveolus
5	Anterior edge of the palatine foramen
6	Meeting point between the M^3^ hypoflexus and alveolus
7	Meeting point of the basiosphenoid and basioccipital on the midsagittal plane
8	Midsagittal border of the foramen magnum
9	Most posterior point of the occipital condyle
10	Suture where the tympanic and occipital meet (posterior edge of the tympanic bulla, between paraoccipital and mastoid processes)
11	Most lateral point of the suture between the tympanic and squamosal

### Analyses

Generalized least squares Procrustes superimposition was used to scale, rotate, and align landmark coordinate sets. This allows superimposition of landmarks for each specimen, without altering the configuration they record. Following superimposition, a consensus configuration of landmarks was computed, which represents the average shape of all specimens analyzed. The consensus was then used to generate partial warp scores, which represent localized shape differences in individual landmark configurations. Uniform components of shape variation record global variations in shape (e.g., shearing) and are also generated by comparing individual configurations to the consensus. Shape differences were modeled using thin-plate splines, which display a deformation grid representing the bending of the consensus configuration of landmarks to a specimen's configuration.

To analyze the data set following superimposition, we used relative warp analysis (RWA). RWA is similar to principal components analysis, but uses partial warp scores as variables and weights the resultant components by their bending energy (the energy needed to bend the consensus to a target configuration). Superimposition and RWA for each view of the skull were performed using the tpsRelw program [56]. Following RWA, partial warp scores, uniform components, and relative warp scores were saved for subsequent analyses.

Partial warp scores and uniform components were then used as variables in a stepwise canonical variates analysis (CVA), which was performed in SPSS 15.0. CVA uses the differences between groups to compute a set of canonical variate scores, to determine which linear combinations of variables best separate groups. Species were used as *a priori* groups in the analysis and the classification phase of the analysis was used to test the ability of the canonical variate scores to classify species. The partial warp scores and uniform components were then regressed onto the canonical variate scores using the tpsRegr program (version 1.28) [57]. This allowed visualization of the skull shapes associated with combinations of canonical variate scores.

To examine the phenetic affinities of the species studied, the partial warp scores were also used as variables in a hierarchical cluster analysis. Cluster analysis using unweighted pair-group method with arithmetic mean (UPGMA) was performed in SPSS 15.0.

## Results

### Relative Warp Analysis

**Figure 3:**
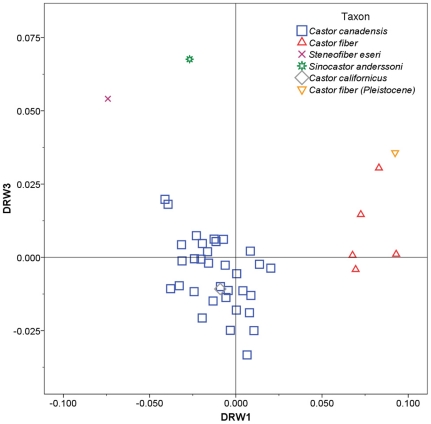
Relative warp plot for the dorsal aspect of the skull. Skull shapes associated with each axis are indicated in Figure 4.

**Figure 4:**
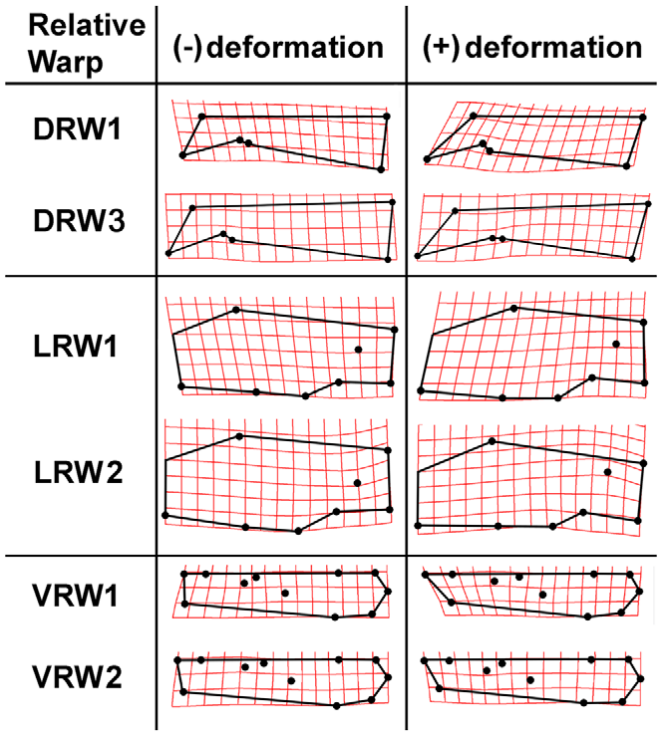
Thin-plate splines indicating the maximum observed deformations of the skull along each relative warp axis.

Separate RWA were run for each view of the skull. RWA for the dorsal aspect of the skull yielded three significant warps (eigenvalues >1.0) explaining 81.7% of the observed variation in shape. Of these, dorsal relative warps 1 and 3 showed separation of beaver species (Fig. 3). DRW1 explained 52.6% of variation and showed good separation of *Castor fiber* (positive scores) from the other species (with scores near 0 or negative). Positive DRW1 scores are associated with relatively elongate nasals (Fig. 4). DRW3 explained 14.4% of the variance and separated *Sinocastor* and *Steneofiber* (positive scores) from all *Castor* species (with less positive or negative scores). Positive DRW3 scores are associated with a posteriorly located orbit and postorbital process (Fig. 4).

**Figure 5:**
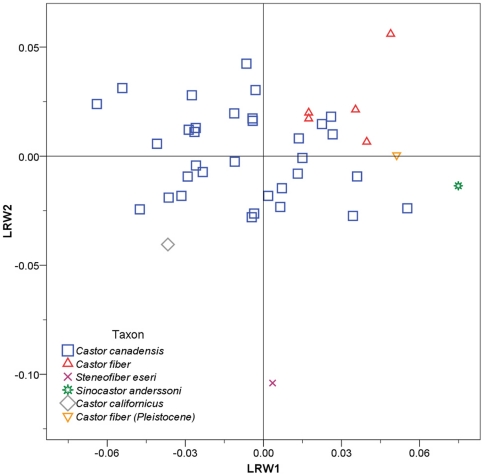
Relative warp plot for the lateral aspect of the skull. Skull shapes associated with each axis are indicated in Figure 4.

Relative warp analysis for the lateral aspect yielded five significant warps explaining 85.1% of variation. Lateral relative warps 1 and 2 showed some separation of species (Fig. 5). LRW1 explained 32.1% of the variance, *Castor fiber* and *Sinocastor* had positive scores while *C. canadensis* had a wide range of values. Positive LRW1 scores are associated with relatively elongate nasals (Fig. 4). LRW2 accounted for 22.5% of the variance and separated *Steneofiber*, with highly negative scores, from *Castor* and *Sinocastor*. Positive LRW2 scores are associated with a relatively elevated external auditory meatus (Fig. 4).

**Figure 6:**
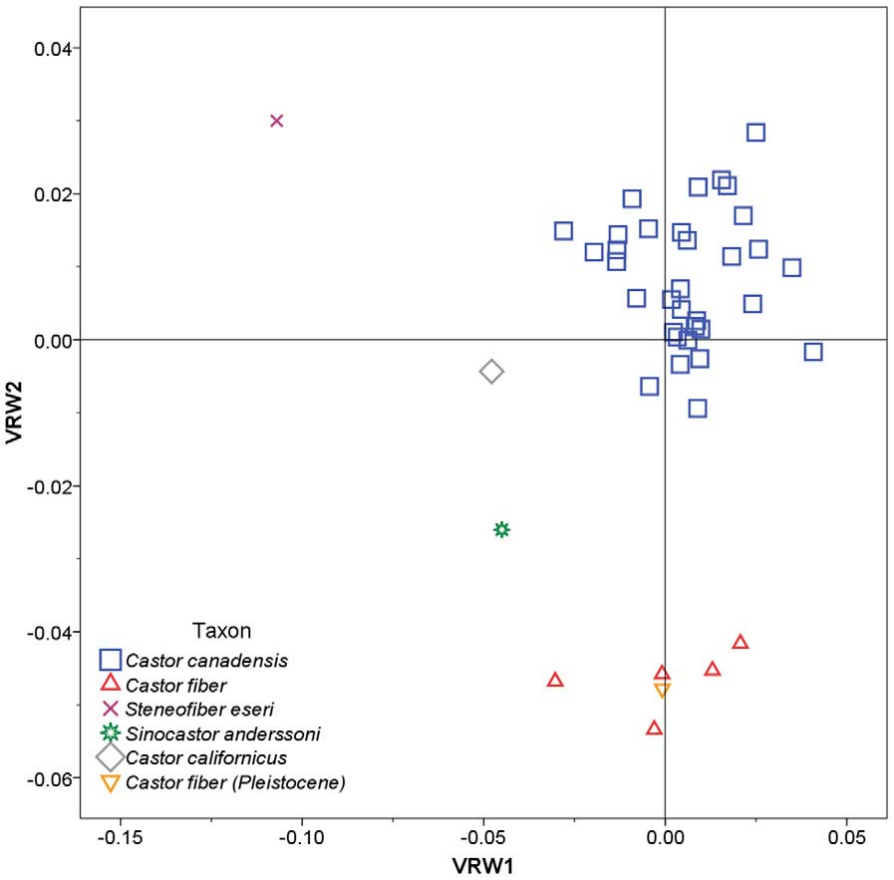
Relative warp plot for the ventral aspect of the skull. Skull shapes associated with each axis are indicated in Figure 4.

Relative warp analysis for the ventral aspect yielded eight significant warps explaining 91.1% of variation. Ventral relative warps 1 and 2 showed good separation of most species (Fig. 6). VRW1 explained 33.3% of the variance and separated *Steneofiber*, with highly negative scores, from all other species. Negative VRW1 scores are associated with a relatively shorter tooth row and a smaller distance between the upper P4 and infraorbital foramen (Fig. 4). VRW2 explained 25.5% of the variance and showed good separation of most species; *Castor fiber* had highly negative scores, *Sinocastor* had intermediate negative scores, *C. canadensis* and *C. californicus* had scores near 0, and *Steneofiber* had positive scores. Negative VRW2 scores are associated with an elongate incisive foramen and posteriorly located palatine foramen (Fig. 4).

The two extant beavers, *C. canadensis* and *C. fiber*, overlap little in the RWA. The Pleistocene specimen of *C. fiber* groups closely with extant *C. fiber* in all analyses, while the extinct *C. californicus* falls within or near the values of extant *C. canadensis*. The extinct *Sinocastor* and *Steneofiber* fall outside of the observed ranges for *C. canadensis* and *C. fiber* in all analyses.

### Canonical Variates Analysis

**Figure 7:**
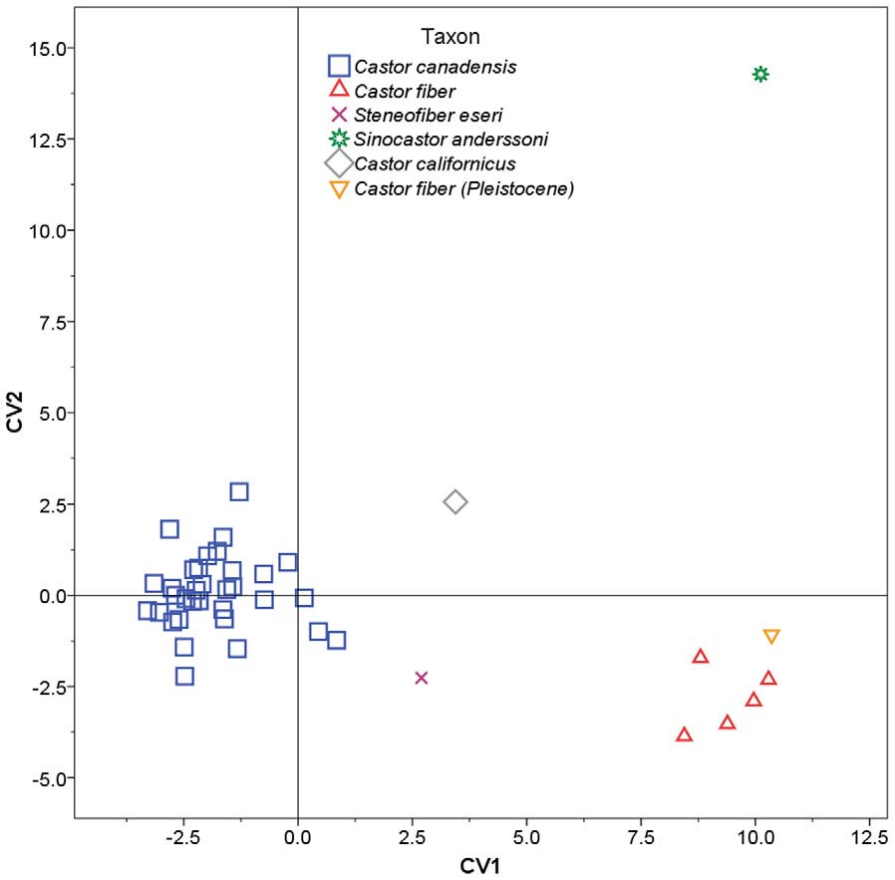
Plot of the first and second canonical variate scores. Skull shapes associated with each axis are indicated in Figure 9.

**Figure 8:**
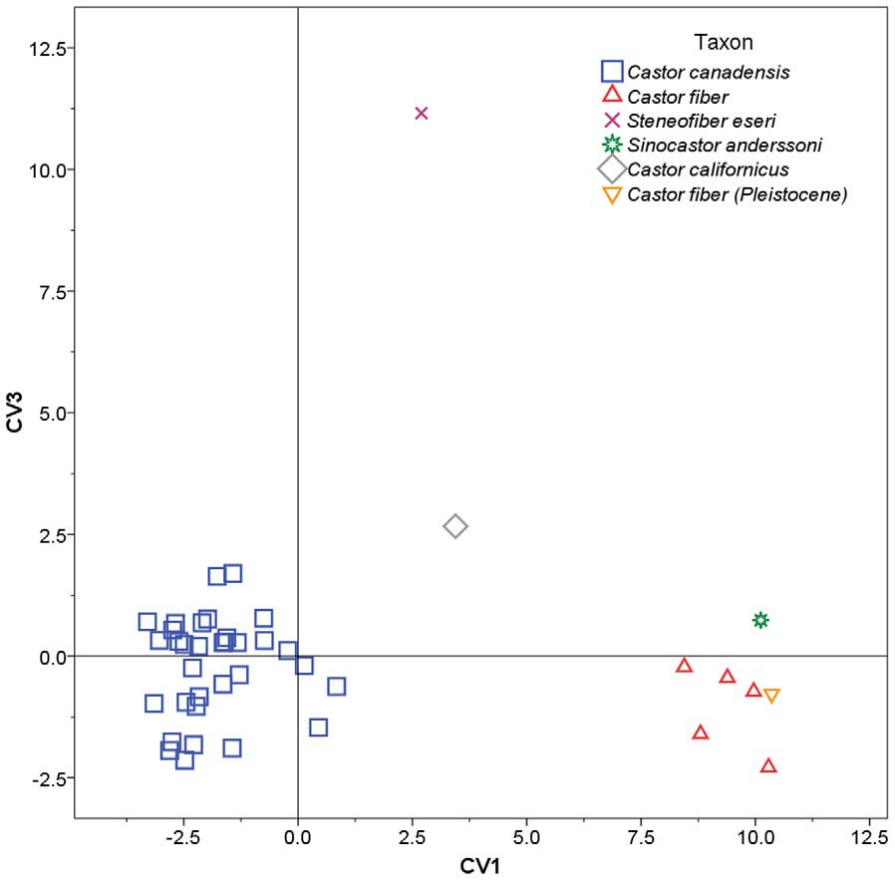
Plot of the first and third canonical variate scores. Skull shapes associated with each axis are indicated in Figure 9.

Stepwise CVA was performed using partial warp and uniform component scores as variables, and individual species acting as *a priori* categories (Table 2). The stepwise model included 11 of 16 partial warps and showed significant separation of groups (Wilks' λ = 0.001, F_(1, 36)_ = 19.448, *p* = 9.438×10^−26^). The analysis yielded three canonical variates with significant discriminating power, accounting for a total of 100.0% of variance in the data set (Figs. 7, 8).

**Table 2:** Summary statistics for canonical variates analysis of beaver species.

	CV1	CV2	CV3
Eigenvalue	18.260	6.946	3.664
% Variance Explained	63.2	24.1	12.7
Wilks' λ	0.001	0.027	0.214
X^2^	206.977	113.799	48.509
Canonical Correlation	0.974	0.935	0.886

**Figure 9:**
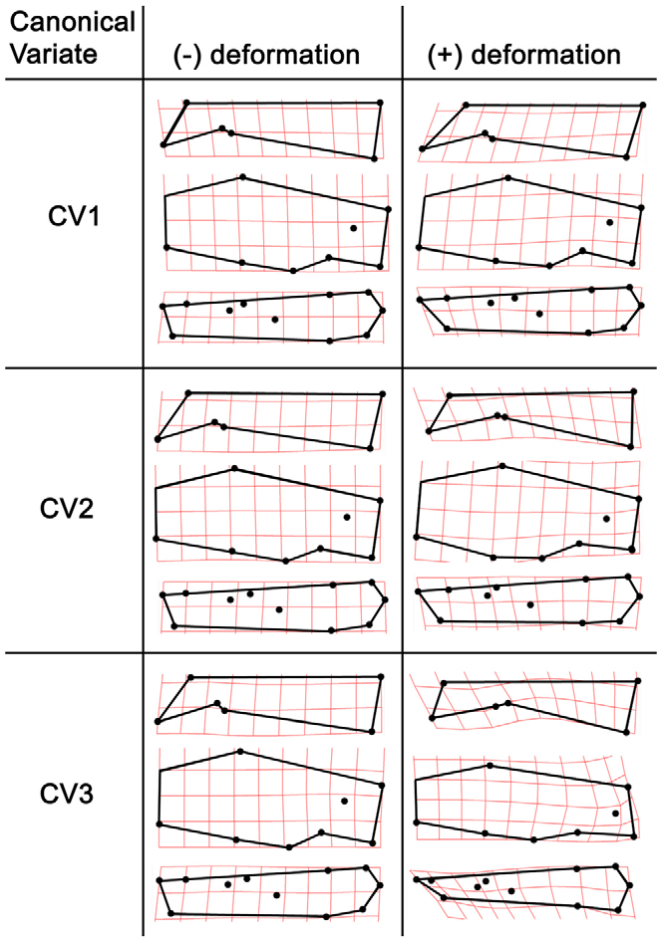
Thin-plate splines indicating the maximum observed deformations of the skull along each canonical variate axis.

Canonical variate 1 (CV1) accounted for 63.2% of the variance and showed good separation of taxa (Fig. 7). *Castor canadensis* had negative or near 0 scores, *C. fiber* (extant and Pleistocene) and *Sinocastor* had high positive scores, while *C. californicus* and *Steneofiber* had intermediate scores. Positive CV1 scores are associated with relatively elongate nasals, anteriorly positioned and larger incisive foramen, and a posteriorly positioned palatine foramen (Fig. 9). CV2 accounted for 24.1% of the variance and primarily separated *Sinocastor*, with high positive scores, from all other taxa (Fig. 7). To a lesser degree CV2 also separated *C. canadensis* and *C. californicus*, with positive and near 0 scores, from *C. fiber*, with negative scores. Positive CV2 scores are associated with narrow interorbital region and posteriorly positioned orbit, smaller postorbital process, shallower nuchal region, inferiorly shifted external auditory meatus, posteriorly positioned infraorbital foramen, and shorter cheek tooth row (Fig. 9). Negative CV2 scores are associated most strongly with a broad orbital distance and larger postorbital process. CV3 accounted for 12.7% of the variance and separated *Steneofiber* from all other taxa (Fig. 8). *Steneofiber* had high positive scores, while all other taxa had low positive or negative scores. Positive CV3 scores are associated with a posteriorly shifted orbit, smaller postorbital process, shallower skull, inferiorly and posteriorly shifted external auditory meatus, anteriorly slanted occipital, smaller incisive foramen, and shorter and anteriorly shifted cheek tooth row (shorter rostrum/longer braincase) (Fig. 9).

The classification phase of the analysis was used to test the ability of the model to separate individuals into species. The classification of individuals based on their original groups was 100% correct, while 95% were correctly classified when cross validated (where individuals are excluded from creating the model and classified using the remaining individuals). *Sinocastor* was classified as *Castor fiber* when cross validated, whereas *Steneofiber* was classified as *C. canadensis*. In both cases the classifications fall outside the range of values for the other species and showed low conditional probabilities, suggesting the classification was likely incorrect.

### Cluster Analysis

**Figure 10:**
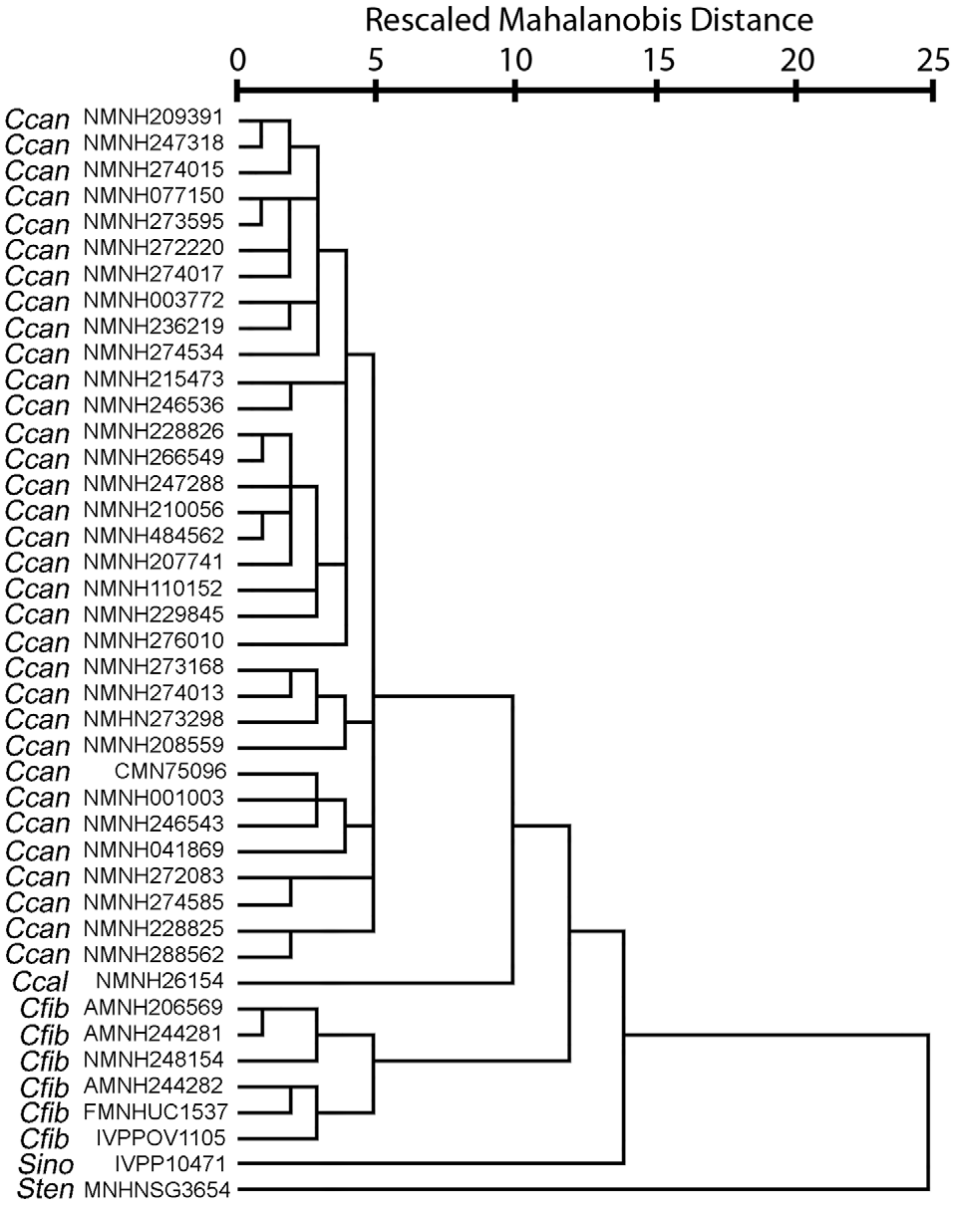
Phenogram of beaver species. Phenogram produced by hierarchical cluster analysis of partial warp and uniform component scores.

Hierarchical cluster analysis, using partial warp and uniform component scores as variables, consistently grouped individuals with members of their own species (Fig. 10). All individuals of the extant North American beaver, *Castor canadensis*, clustered together. This cluster of *C. canadensis* was sister to the extinct *C. californicus*, also of North America. European and Asian specimens of *C. fiber* grouped together, including a specimen from the Pleistocene of England. *Sinocastor* appears as sister to the *Castor* clade, with *Steneofiber* falling outside of this cluster.

### Comparative Description

#### Cranium

**Figure 11:**
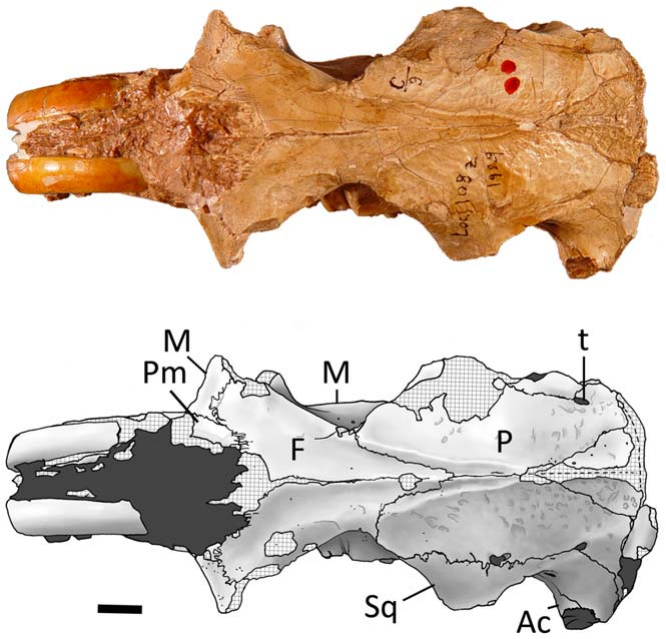
Dorsal view of *Sinocastor anderssoni* skull specimen, IVPP v-10471. Abbreviations: Ac: acoustic meatus; F, frontal; M, maxilla; P, parietal; Pm, premaxilla; Sq, squamosal; t, temporal foramen. Scale bar represents 1 cm.

**Figure 12:**
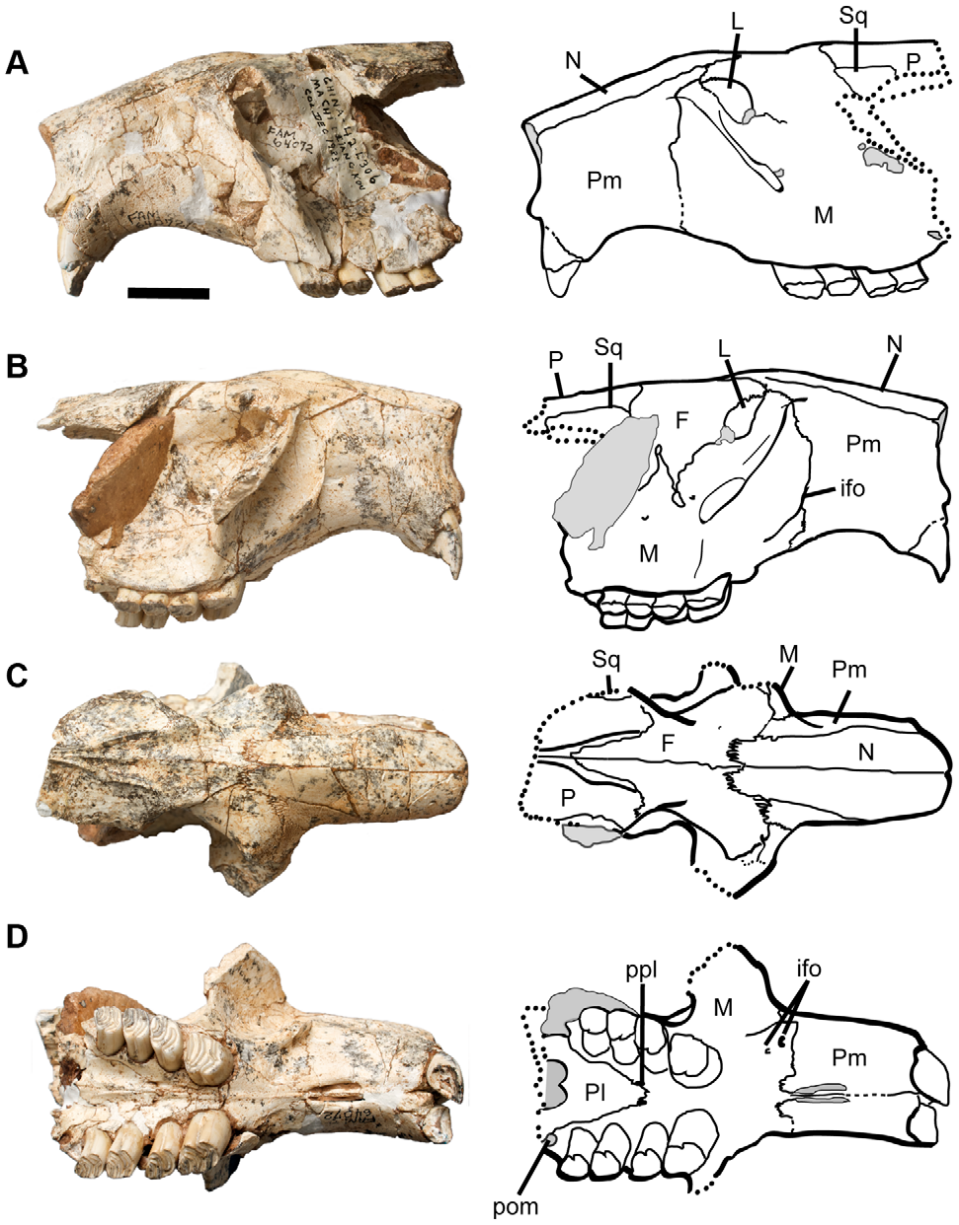
*Sinocastor anderssoni* partial skull, FAM 64072. Abbreviations: F, frontal; ifo, infraorbital foramen; L, lacrimal; M, maxilla; N, nasal; P, parietal; Pl, palatine; Pm, premaxilla; posterior maxillary foramen; Sq, squamosal. Scale bar represents 2 cm.

Dorsal view (Figs 11, 12) — In overall proportions, the cranium of *Sinocastor* (IVPP v-10471) is more elongate than that of *Castor*. The nasals extend posteriorly to a point posterior to the anterior margin of the orbits, similar to *C. fiber*. In *C. canadensis* the nasals are relatively shorter, ending just posterior to the orbital rim. The posterior extent of the premaxillaries is anterior to that point, similar to the condition in *Castor*. The maxillary contact with the frontals is longer in *Sinocastor* than in *Castor*. In *C. canadensis* this contact is very short. The jugal is lacking in IVPP v-10471 and IVPP v-10472 but enough of the maxilla is preserved to show that the jugal-maxillary contact was more posterior in *Sinocastor* than in *Castor*. The frontals extend posteriorly and form a V-shaped suture with the parietals that extends about half the length of the neurocranium, similar to the condition in *Castor*. In *Sinocastor*, the postorbital constriction is greater and the frontals taper posteriorly much more dramatically than in *Castor*. Parasagittal crests originate above the orbits and converge posteriorly to form a single sagittal crest at the posterior limit of the parietals. Dorsal to the orbits in *Sinocastor* are low ridges that merge posteriorly with the anterior end of the parasagittal crests. These supraorbital ridges are not coincident with the dorsal margin of the orbit, but are set medial to it and are curved. This type of ridge is generally lacking in *Castor canadensis*, but a similar ridge is present in specimens of *C. fiber*. The parietals of *Sinocastor* have a rugose surface, typical of all castorids. Along the parietal-squamosal suture is a single temporal foramen on each side, situated posterior to the glenoid fossa. In *Castor* there is usually a variable number of much smaller foramina all along the suture. The neurocranium of *Sinocastor* appears more elongate than that of *Castor*.

A triangular interparietal bone is present, which is longer (anteroposteriorly) than wide. The sagittal crest forms at the midline, and posteriorly, the sagittal crest joins the occipital crest. In *Castor*, the parasagittal and sagittal crests are variable in shape and height, which appears to be related to ontogenetic age.

**Figure 13:**
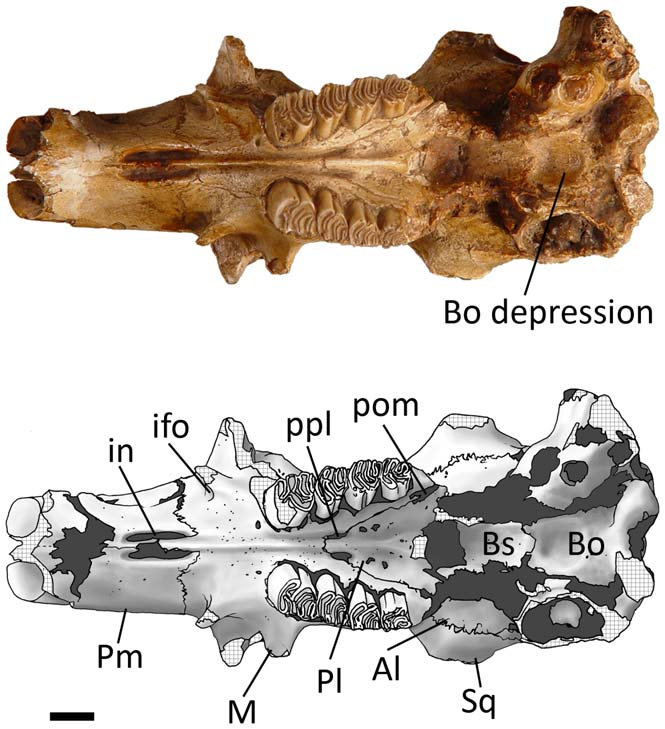
Ventral view of *Sinocastor anderssoni* skull specimen, IVPP v-10471. Abbreviations: Al, alisphenoid; Bs, basisphenoid; Bo, basioccipital; ifo, infraorbital foramen; in, incisive foramen; M, maxilla; Pl, palatine; Pm, premaxilla; pom, posterior maxillary foramen; ppl, posterior palatine foramen; Sq, squamosal. Scale bar represents 1 cm.

Ventral view (Figs 12, 13) — The rostrum of *Sinocastor* is more elongate than that of *Castor*. The incisive foramina are also relatively larger in *Sinocastor* than in *Castor*. The premaxillary-maxillary suture runs perpendicular to the centerline of the diastema and crosses the centerline at the posterior end of the incisive foramina, as in *Castor*. The paired grooves on the palate of *Castor* and most other castorids are also present on *Sinocastor* running from the palatine-maxillary suture on the palate to the posterior end of the incisive foramina. In *Castor* there are usually ridges lateral to these grooves that parallel the midline, between the cheek teeth and the incisive foramina. These lateral ridges are sometimes as high as the central ridge between the tooth row and the incisive foramina in *Castor*. In *Sinocastor*, the central ridge is very high and the lateral ridges are almost completely absent.

In both *Castor* and *Sinocastor*, the tooth rows strongly diverge posteriorly to the same degree. The maxillary-palatine suture extends anteriorly to a point even with the anterior margin of M1 on both *Castor* and *Sinocastor*. The posterior palatine foramina are along the maxillary-palatine suture even with the boundary between M1 and M2. There are several smaller foramina within the palatine posterior to the posterior palatine foramen in both *Castor* and *Sinocastor*. The posterior maxillary foramen is small and circular, positioned posterior to M3 in both beavers. In *Castor* this foramen is variable, being sometimes very small or in other cases slit-like.

Due to breakage, the region of the ventral skull posterior to the toothrows is incompletely known. The posterior edge of the palate and the pterygoids are lacking. The edges of the foramen ovale are not preserved, but the area in which it occurs is filled with matrix suggesting its location is at the anterior border of the bulla, similar to that of *Castor*. Between the bullae of *Sinocastor* is a shallow, circular depression. Along the posterior margin of this depression is a low ridge that extends posteriorly for about 5 mm and joins the base of the foramen magnum between the occipital condyles. The depression is much deeper in *Sinocastor* than in *Castor*. Also, unlike *Sinocastor*, the depression in *Castor* extends posteriorly to the base of the foramen magnum and the sides of the depression are steep, forming distinct walls laterally and posteriorly and creating a nearly rectangular outline.

The bulla in *Sinocastor* is similar in shape to that of *Castor*. It is rounded ventrally and the external auditory meatus is a long, thick tube that extends dorsolaterally. There is a low ridge along the tube in *Sinocastor* that runs along its posterior margin to the base of the external opening. A similar ridge is present in *Castor* but is much more pronounced, forming a broad flange.

**Figure 14:**
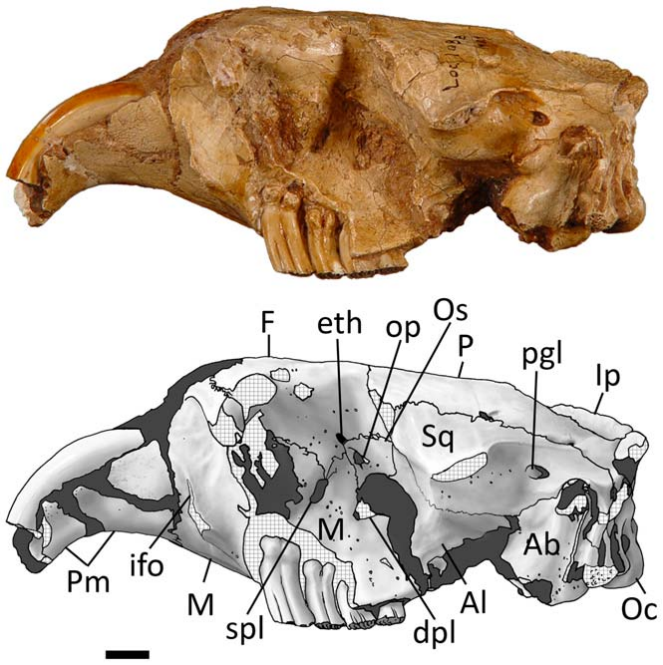
Left lateral view of *Sinocastor anderssoni skull*, IVPP v10471. Abbreviations: Ab, auditory bulla; Al, alisphenoid; dpl, dorsopalatine foramen; eth, ethmoid foramen; F, frontal; ifo, infraorbital foramen; Ip, interparietal; M, maxilla; Oc, occipital condyle, op, optic foramen; Os, orbitosphenoid; P, parietal; pgl, postglenoid foramen; Pm, premaxilla; spl, sphenopalatine; Sq, squamosal. Scale bar represents 1 cm.

Lateral view (Figs 12, 14) — The *Sinocastor* skull is sciuromorphous with a steeply tilted zygomatic plate. The premaxillary-maxillary suture runs almost directly vertically on the side of the rostrum. The infraorbital foramen is small and slit-like, posterior to the premaxillary-maxillary suture, low on the side of the skull, just above the diastema. Lateral to it is a flange for the attachment of the superficial masseter. All of these features do not differ from those of *Castor.*

Anterior to the zygomatic plate in *Sinocastor* is the fossa for the attachment of the anterior lateral masseter. The anterior border of this fossa starts at the flange for the superficial masseter and is manifest as a low ridge that runs dorsally but fades at the point where it crosses the premaxillary-maxillary suture. This is different in specimens of *Castor*; where the anterior boundary of the masseteric scar tends to be a high ridge all the way to the dorsal margin of the skull and is tilted anteriorly. The premaxillary-maxillary suture crosses this anterior ridge just above its center. In *Sinocastor* the ridge disappears before the suture crosses the anterodorsal corner of the depression.

The zygomatic arches are completely lacking in the *Sinocastor* skulls, except for FAM 64072, on the right side, where the anteriormost part of the zygomatic arch, just anterior to the orbit, is partially preserved. The jugal is lacking, but this specimen shows that the contribution of the maxilla (in lateral view) to zygomatic plate appears similar to the condition seen in *C. canadensis*. In *C. fiber* the maxilla appears relatively thicker. The orbital wall in IVPP v-10471 is very well preserved. The lacrimal foramen in the anterodorsal corner of the orbital wall is larger than in *Castor*. The optic foramen is small and dorsal to the center of the orbital wall (above M2) within the orbitosphenoid bone. Anterior and ventral to it is the sphenopalatine foramen (above M1). It is within the maxillary bone. It is larger than the same foramen in *Castor* and oval in shape. Just anterior and dorsal to the optic foramen is a small ethmoid foramen, just above the frontal-orbitosphenoid suture. Dorsal to the ethmoid foramen is a minute frontal foramen, the presence of which is variable in *Castor*. Posterior to the sphenopalatine foramen (above M3) is a large, circular interorbital foramen. Ventral to it (above the M2-M3 boundary) is a smaller dorsal palatine foramen. These foramina are nearly identical to that in *Castor*. A large sphenoidal fissure opens posterior to the interorbital foramen, bounded laterally by the alisphenoid. Along the dorsal surface of the fissure, in the alisphenoid, are two small grooves that extend anterodorsally. In *Castor* these same two grooves originate in two small foramina, one just anterior to the other; these are interpreted as the buccinator (more dorsally located) and the masticatory foramina. The most ventral part of the maxillary-alisphenoid suture is well posterior to the tooth row. In *Castor* this suture is much more anteriorly located, dorsal to M3. The ventral floor of the orbital wall in the specimens of *Sinocastor* is a smooth surface that slants laterally. In *Castor* the floor of the orbit can be comprised of a series of mounts of bone that encapsulate the bases of the teeth. However, these are variable in size. In younger individuals, they are very high and appear to be reduced in size in later ontogenetic age. In the very oldest individuals of *Castor* observed (teeth almost completely worn to the base) the area is smooth as in *Sinocastor*. The presence of these mounds of bone, or tooth capsules in *Castor* is associated with the greater crown-height of the cheek teeth. In IVPP v-10471 the teeth do not appear heavily worn; suggesting that the individual is not in old age, yet there is no indication of the cheek tooth capsules in the floor of the orbit. Posterior to the glenoid fossa is a large, oval postglenoid foramen, slightly larger than in *Castor* where the foramen is usually circular.

Posterior View— The occipital is nearly vertical in *Sinocastor* and the skull appears to be slightly less broad than that of *Castor*. The mastoid exposure on the back end of the skull is roughly triangular, extending closest to the centerline of the occipital at its most dorsal point. The mastoid foramen is along the occipital-mastoid suture at the apex of the mastoid triangle, as in *Castor*. An additional foramen is present on the skull of *Sinocastor* and *Castor* along the lateral edge of the mastoid, just ventral to the level of the external meatus of the auditory bulla. Any indication of the paroccipital processes is lost on the *Sinocastor* specimen due to breakage.

#### Dentition

**Figure 15:**
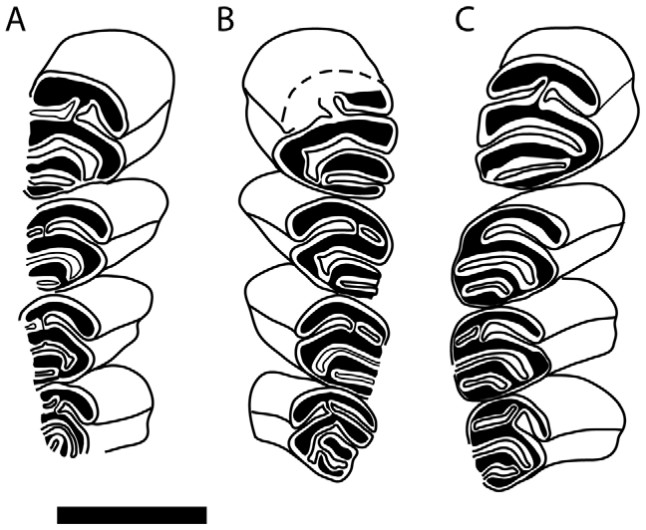
Occlusal pattern of upper cheek teeth of *Sinocastor*. IVPP v-10471 right tooth row (A), left tooth row (B), and FAM 64072 right tooth row (C), are shown with anterior end of the tooth row toward the top of the page. Scale bar represents 1 cm.

The dentition of *Sinocastor* has been previously described in detail elsewhere (Teilhard de Chardin, [29]). Young [26] used incisor shape and the occlusal morphology of the upper premolar to distinguish *Sinocastor* from *Castor* (see Historical Review above). The cross sectional incisor shape of *Castor* was found to differ from that of *Sinocastor*, with that of *Sinocastor* being rounded relative to the more triangular shape of *Castor*. However we did not find the occlusal morphology of the upper premolar to be distinguishing between the two genera. Specifically, Young's [26] observation of the anterior flexi of P4 alternating in *Sinocastor* is not supported by the available specimens (see Fig. 15). In all cases, the paraflexus and hypoflexus meet in the center of the tooth and are not alternating.

The greatest difference in the cheek teeth of *Sinocastor* and *Castor* is the crown height and the depth of the striae. In *Sinocastor* all of the cheek teeth are rooted, and the bases of the roots form small nubs (Fig. 14). In *Castor*, including the Tertiary species from North America, *C. californicus*, the cheek teeth do not show well-developed roots, and usually the root-ends are smoothly rounded see [30], [58]. However, small roots may sometimes occur in *Castor*, as they have been reported in the Pliocene *C. californicus* [59]. The total height of the crowns of *Sinocastor* cannot be measured because there were no juvenile individuals available with unerupted cheek teeth. However, in comparably aged specimens of *Castor* the cheek teeth are much higher crowned (see also above for description of orbital floor in lateral view.).

Compared to *Castor*, the buccal striae of the upper cheek teeth are shorter in *Sinocastor*. In all *Castor* specimens, these striae extend to the base of the crown. The absence of striae is manifested on the occlusal surface of the cheek teeth by the closure of the flexi into fossettes. Only in the most senile specimens of *Castor* do any of the flexi close off into fossettes. On IVPP v-10471, some of the bone has been removed from the lateral side of the left tooth row, exposing striae that do not extend to the base of the crown. Also, in AMNH 64072, a non-senile adult individual (Fig. 12), some of the flexi have become fossettes. Thus the suggestion of Young [22] (See Historical review) that the striae of the cheek teeth probably reached the base of the crown in *Sinocastor* is not supported [26].

In summary, dentally, *Sinocastor* differs from *Castor* in the cross-sectional shape of the incisors, the relatively lower crown height and shorter striae (as noted by Teilhard de Chardin [31]), as well as the presence of prominent roots on the cheek teeth. There are also a number of differences in the morphology of the skull that separate *Sinocastor* from *Castor* (see the revised diagnosis below).

### Revised diagnosis

Order Rodentia Bowdich, 1821

Family Castoridae Hemprich, 1820

Subfamily Castorinae Hemprich, 1820

*Sinocastor* Young, 1934

Castorine, slightly larger and more robust than *Castor*; occlusal pattern on cheek teeth similar to that of *Castor* (three persistent buccal flexi and one lingual on upper cheek teeth, three persistent lingual flexids and one buccal on lower cheek teeth). Dental characters distinctive from *Castor*: cheek teeth subhypsodont (roots form on all cheek teeth), lower than in *Castor*; buccal striae on upper cheek teeth and lingual striids on lower cheek teeth do not extend the entire height of the crown, thus occlusal flexi (-ids) become fossettes (-ids) in late stages of wear. Cranial characters distinctive of *Castor* are listed below and those with an asterix (*) are supported by morphometric analysis: 1)* more elongated rostrum, 2) * relatively longer incisive foramina, 3) weaker development of the anterior margin of the masseteric scar on the rostrum; 4) shape of the maxillary-frontal contact on the dorsal side of the skull in antorbital region; 5) * greater development of the postorbital constriction; 6) * neurocranium not as broad, more elongated; 7) Single temporal foramen; 8) basioccipital recess smaller, rounded, shallower; 9) lateral ridge on external meatus of the bulla less pronounced; 10) palatal midline ridge anterior to tooth row higher than lateral ridges; 11) * more posterior palatine foramen; 12) ventral maxillary-alisphenoid suture posterior to tooth row; 13) floor of orbit smooth (no capsules for cheek teeth roots).

## Discussion

This study uses a geometric morphometric approach to compare a nearly complete skull of the fossil castorid *Sinocastor*, IVPP v-10471, (Late Miocene China) to a sample of modern *Castor* skulls (N = 38), representing *C. fiber*, *C. canadensis*. Also included were a Pleistocene specimen of *C. fiber* and a specimen of the North American fossil taxon *C. californicus*. The results of the study suggest that IVPP v-10471 falls outside the range of variation observed for *Castor*. We therefore favor that the specimen be retained in the genus *Sinocastor*, and considered *S. anderssoni*.

When the genus *Sinocastor* was first defined by Young [26], this was done on the basis of two dental and two cranial characters (see Historical review). Of the two dental characters only the cross sectional shape of the incisor appears helpful for distinguishing *Castor* and *Sinocastor*. However as discussed above (See Dentition), there are some other important dental differences: As noted by Teilhard de Chardin [29], *Sinocastor* differs from *Castor* in that it has shorter striae(ids) and prominent roots on the cheek teeth. The cranial characters noted by Young [26] were the presence of masseteric fossa and a very narrow post-orbital constriction. Here we would add the presence of a shallow basioccipital depression. The morphometric analysis presented here brings to attention several cranial differences, some of which were not noted in earlier studies. Compared to *Castor, Sinocastor* exhibits: 1) increased post-orbital constriction of the skull; 2) basioccipital depression that is shorter in the anterior posterior direction; 3) lengthened incisive foramen; 4) more posterior palatine foramen; 5) longer rostrum; and 6) longer braincase.

**Figure 16:**
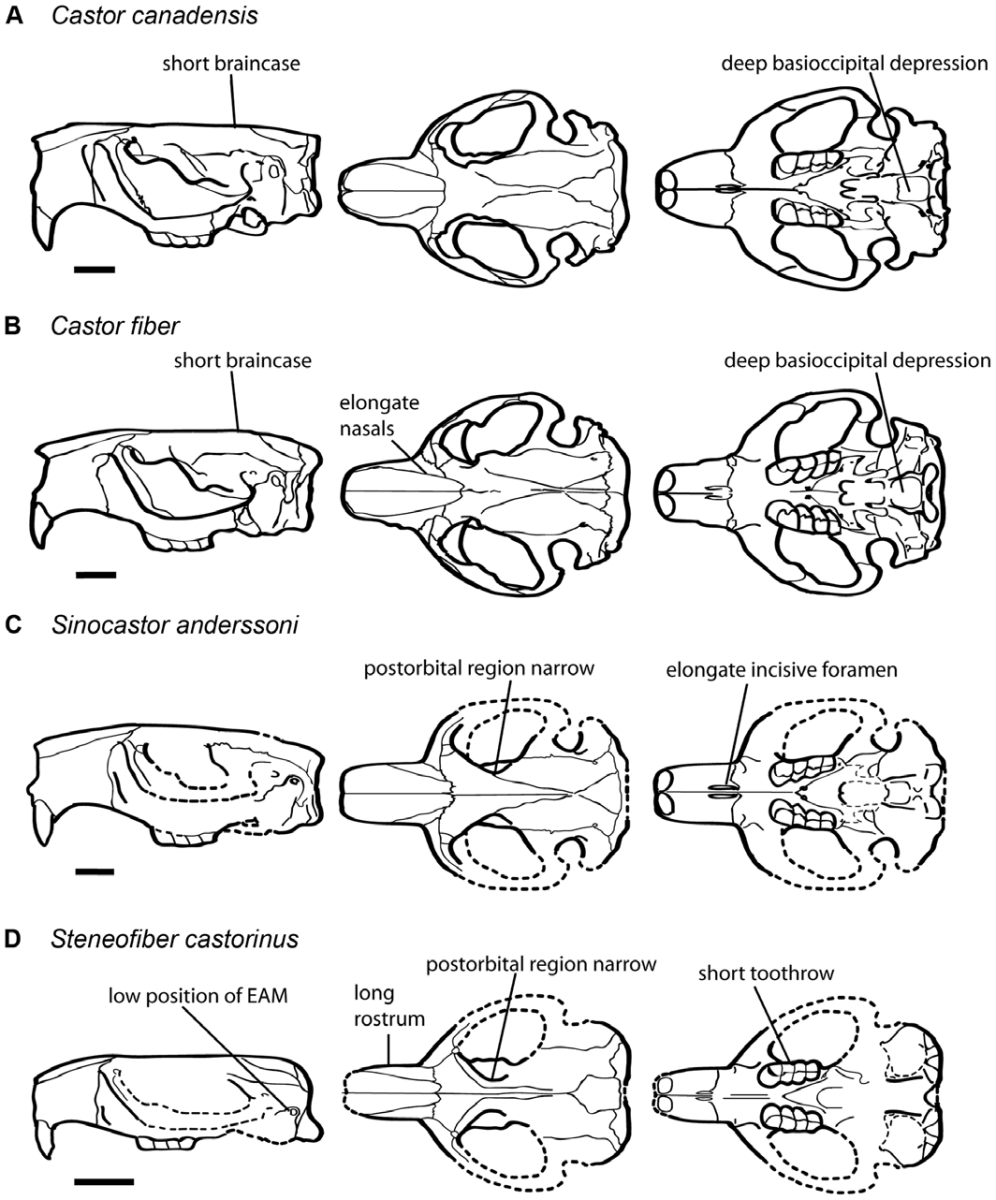
Comparison of *Castor*, *Sinocastor* and *Steneofiber* skulls illustrating major skull shape differences. Scale bar represents 2 cm.

The analysis also included a specimen of the early Miocene European castorid, *Steneofiber castorinus*, and the Pliocene *C. californicus* of western North America. A cluster analysis including extant *Castor* and the three fossil taxa found *C. canadensis + C. californicus* and *C. fiber* to form a group, with *Sinocastor* and *Steneofiber* forming successive outgroups (Fig. 10). *Steneofiber* provides a model for the primitive condition, and shows that features seen in *Sinocastor* such as the presence of increased post-orbital constriction, longer rostrum and longer braincase are relatively primitive (Fig. 16).

Previously it has been suggested that *Castor* evolved from *Chalicomys* [60], *Chalicomys* has been considered to be represented in the fossil record from the middle through latest Miocene (MN 5-13) of Europe [13]. However, a more recent study suggests *Chalicomys* is restricted to MN 9/10 [16], with older fossils being attributed to *Steneofiber*. *Chalicomys* is primarily represented by dental remains, which are generally *Castor*-like, but with shorter striae/iids on the cheek teeth. The striae/iids of *Chalicomys* are also shorter than those of *Sinocastor* [13], suggesting that lengthened striae/iids may be an autapomorphy of *Sinocastor* + *Castor*. The phylogenetic hypothesis that *Chalicomys* is sister to *Sinocastor* + *Castor* should be further evaluated. Unfortunately, cranial material is not known from *Chalicomys*. Another taxon that might be informative is the near relative, *Steneofiber depereti*. *S. depereti* (MN 3–MN 7/8) is older than most records of *Chalicomys* but is dentally very similar, such that in some cases it is difficult to distinguish the two taxa [16], [61]. *S. depereti* is represented by cranial material including two partial skulls collected from Artenay (France) (Ar 2529, 283) housed at the Museum National D'Histoire Naturelle (Paris). Comparative analysis of *S. depereti* with *Sinocastor + Castor* may be very helpful for elucidating the early evolution of the lineage that ultimately gave rise to *Castor*.

If *Sinocastor* is sister to *Castor*, this would suggest that their common ancestor may have lived in East Asia, perhaps derived from a *Chalicomys*-like ancestor that had dispersed from Europe to East Asia in the Late Miocene. From Asia, *Castor* could have dispersed and north eastward to North America, via the Beringian Isthmus, and northwestward into Europe. The oldest *Castor* in Europe may be *Castor neglectus*, a rare form known from 10-12 Ma deposits (MN9) in Germany and Moldavia, which exhibits *Castor* like dentition and is “nearly as hypsodont as *Castor fiber*” [[13], page 289]. Fossil evidence suggests that *Castor* did not flourish in Europe until the beginning of the Pliocene [13]. In North America, the earliest records of *Castor* are from the late early Hemphillian, approximately seven million years ago [14], whereas in East Asia, *Castor* does not appear in the fossil record until the Middle Pleistocene [62]. The late appearance of *Castor* in Asia might be associated with the presence of *Sinocastor* populations, which persisted in China at least until the middle Pleistocene [62]. One factor that may have allowed *Sinocastor* to persist is that its population was semi-isolated from its European near-relatives by the Himalayan Mountain belt and associated arid zone [62].

For lineages with living representatives it is sometimes possible to use molecular evidence to reconstruct evolutionary histories. Unfortunately, molecular evidence relating to the origins of *Castor* remains ambiguous as to the most likely region of origin. Phylogeographic investigation using mitochondrial (mtDNA) of relict *C. fiber* populations, recovered two evolutionary groups of *C. fiber*, an Eastern Europe + Asia and Western Europe population [63]. Neither could be distinguished as ancestral to the other. It is likely that the distribution of relict *C. fiber* populations reflects Pleistocene glaciation events [63], rather than any phylogeoegraphic patterns associated with the earliest phases of *Castor* evolution. In the end, perhaps the best approach is to answer questions relating to the origins of *Castor*, is to focus on the fossil record. Fossil evidence of *Castor* from Europe and North America, both cranial and postcranial remains, would help fill in the details of the story, particularly the timing and associated environmental changes. Reconstructing the evolutionary history of the group could also be helped by including evidence from ancient molecules, such as DNA, from fossil taxa. The Eurasian record of *Castor* includes various forms whose relationships and taxonomy remain to be verified, including the *Castor fiber* near-relatives *C. tamanseis* [64] and *C. praefiber* [65], and the rare, enigmatic *C. neglectus*, which may in fact be only a distant relative of the *Castor*-*Chalicomys* group [64], [65]. Understanding the evolutionary history of *Castor* will require comparison of these fossil forms with *C. canadensis* and *C. fiber*, and also *Sinocastor*.

## Supporting Information

Table S1 List of beaver specimens used in morphometric analyses. CMN =  Canadian Museum of Nature, NMNH  =  Smithsonian Institution (U.S. National Museum of Natural History), AMNH  =  American Museum of Natural History, IVPP  =  Institute of Vertebrate Paleontology and Paleoanthropology, FMNH  =  Field Museum of Natural History, MNHN  =  Muséum National D'Histoire Naturelle.
(0.12 MB DOC)
