# Supplementary material for: Re-Evaluation of Sinocastor (Rodentia: Castoridae) with Implications on the Origin of Modern Beavers
Source: PLoS One. 2010 Nov 15;5(11):e13990. doi: 10.1371/journal.pone.0013990 (PMC2981558; doi:10.1371/journal.pone.0013990)
Supplement: Table S1 — List of beaver specimens used in morphometric analyses. CMN = Canadian Museum of Nature, NMNH = Smithsonian Institution (U.S. National Museum of Natural History), AMNH = American Museum of Natural History, IVPP = Institute of Vertebrate Paleontology and Paleoanthropology, FMNH = Field Museum of Natural History, MNHN = Muséum National D'Histoire Naturelle. (0.12 MB DOC) [file pone.0013990.s001.doc]

**Table S1.** List of beaver specimens used in morphometric analyses. CMN = Canadian Museum of Nature, NMNH = Smithsonian Institution (U.S. National Museum of Natural History), AMNH = American Museum of Natural History, IVPP = Institute of Vertebrate Paleontology and Paleoanthropology, FMNH = Field Museum of Natural History, MNHN = Muséum National D’Histoire Naturelle.

| **Specimen #** | **Genus** | **Species** | **Subspecies** | **Country** | **State/**  **Province** | **Date collected** | **Sex (F/M)** |
| --- | --- | --- | --- | --- | --- | --- | --- |
| CMN 75096 | *Castor* | *canadensis* | ? | Canada | Quebec | Nov. 1976 | M |
| NMNH 001003 | *Castor* | *canadensis* | *mexicanus* | USA | Texas | ? | M |
| NMNH 003772 | *Castor* | *canadensis* | *carolinensis* | USA | Mississippi | 1855 | ? |
| NMNH 041869 | *Castor* | *canadensis* | *texensis* | USA | Texas | 1891 | ? |
| NMNH 077150 | *Castor* | *canadensis* | *sagittatus* | Canada | BC | May, 1895 | F |
| NMNH 110152 | *Castor* | *canadensis* | *canadensis* | Canada | Manitoba | Winter 1900 | F |
| NMNH 207741 | *Castor* | *canadensis* | *missouriensis* | USA | North Dakota | July, 1915 | F |
| NMNH 208559 | *Castor* | *canadensis* | *repentinus* | USA | Arizona | July, 1915 | F |
| NMNH 209391 | *Castor* | *canadensis* | *sagittatus* | Canada | BC | Oct, 1914 | ? |
| NMNH 210056 | *Castor* | *canadensis* | *labradorensis* | Canada | NFLD | 1915 | ? |
| NMNH 215473 | *Castor* | *canadensis* | *sagittatus* | Canada | BC | April, 1916 | M |
| NMNH 228825 | *Castor* | *canadensis* | *sagittatus* | Canada | BC | Feb, 1918 | ? |
| NMNH 228826 | *Castor* | *canadensis* | *sagittatus* | Canada | BC | Feb, 1918 | ? |
| NMNH 229845 | *Castor* | *canadensis* | *missouriensis* | USA | Montana | Aug, 1918 | F |
|  |  |  |  |  |  |  |  |
| NMNH 236219 | *Castor* | *canadensis* | *missouriensis* | USA | Montana | April, 1921 | F |
| NMNH 246536 | *Castor* | *canadensis* | *canadensis* | USA | Pennsylvania | 1926 | ? |
| NMNH 246543 | *Castor* | *canadensis* | *canadensis* | USA | Pennsylvania | April, 1926 | F |
| NMNH 247288 | *Castor* | *canadensis* | *michiganensis* | USA | Michigan | May, 1927 | M |
| NMNH 247318 | *Castor* | *canadensis* | *michiganensis* | USA | Wisconsin | June, 1927 | M |
| NMNH 266549 | *Castor* | *canadensis* | *belugae* | USA | Alaska | April, 1941 | ? |
| NMNH 272083 | *Castor* | *canadensis* | *carolinensis* | USA | Mississippi | Nov, 1941 | M |
| NMNH 272220 | *Castor* | *canadensis* | *idoneus* | USA | Oregon | March, 1942 | ? |
| NMNH 273168 | *Castor* | *canadensis* | *idoneus* | USA | Oregon | July, 1942 | ? |
| NMNH 273298 | *Castor* | *canadensis* | *baileyi* | USA | Oregon | Dec, 1942 | F |
| NMNH 273595 | *Castor* | *canadensis* | *idoneus* | USA | Oregon | June, 1943 | ? |
| NMNH 274013 | *Castor* | *canadensis* | *idoneus* | USA | Oregon | Feb, 1943 | M |
| NMNH 274015 | *Castor* | *canadensis* | *idoneus* | USA | Oregon | Feb, 1943 | M |
| NMNH 274017 | *Castor* | *canadensis* | *idoneus* | USA | Oregon | Feb, 1943 | F |
| NMNH 274534 | *Castor* | *canadensis* | *leucondontus* | USA | Idaho | April, 1923 | ? |
| NMNH 274585 | *Castor* | *canadensis* | *leucondontus* | USA | Idaho | Jan, 1945 | F |
| NMNH 276010 | *Castor* | *canadensis* | *carolinensis* | USA | Georgia | Nov, 1947 | M |
| NMNH 288562 | *Castor* | *canadensis* | *duchesnei* | USA | Utah | ? | F |
| NMNH 484562 | *Castor* | *canadensis* | *carolinensis* | USA | Alabama | Dec, 1972 | F |
| NMNH 248154 | *Castor* | *fiber* | ? | Norway | ? | Oct, 1927 | F |
| AMNH 206569 | *Castor* | *fiber* | *fiber* | USSR | Voronezh Prov. | Oct, 1951 | M |
| AMNH 244281 | *Castor* | *fiber* | *fiber* | USSR | Rhazon Dist. | Nov, 1967 | M |
| AMNH 244282 | *Castor* | *fiber* | *fiber* | USSR | Rhazon Dist. | Nov, 1967 | M |
| IVPP OV1105 | *Castor* | *fiber* | ? | China | ? | ? | ? |
| FMNH UC1537 | *Castor* | *fiber* | ? | England | Cambridge-shire | ? | ? |
| NMNH 26154 | *Castor* | *californicus* | ? | USA | Idaho | ? | ? |
| MNHN SG3654 | *Steneofiber* | *castorinus* | ? | France | Auvergne | ? | ? |
| IVPP 10471 | *Sinocastor* | *anderssoni* | ? | China | Shanxi | 1929 | ? |
